# Oxidation of Hydroxy- and Dihydroxybenzoic Acids Under the Udenfriend's Conditions. An HPLC Study

**DOI:** 10.2174/1874104501812010013

**Published:** 2018-01-31

**Authors:** Mónika Kuzma, Nikoletta Kovács, Lilla Sziva, Gábor Maász, Péter Avar, Pál Perjési

**Affiliations:** 1 Institute of Pharmaceutical Chemistry, University of Pécs, H-7624 Pécs, Rókus str. 2, Hungary; 2 Department of Biochemistry and Medical Chemistry, University of Pécs, H-7624 Pécs, Szigeti str. 12, Hungary

**Keywords:** Udenfriend's system, Hydroxyl radical, Salicylic acid, Dihydroxybenzoic acid, HPLC-UV-Vis, HPLC-ESI-MS

## Abstract

**Background:**

Non-enzymatic hydroxylation of aromatic compounds to the respective phenolic derivatives is a possible metabolic pathway of xenobiotics. The formed metabolites can undergo consecutive oxidative reactions with free radicals to form potential toxic molecules.

**Objective:**

Development of HPLC methods to separate, identify and quantitate the main products formed from salicylic acid, 2,3-dihydroxybenzoic acid and 2,5-dihydroxybenzoic acid under *in vitro* hydroxylation conditions (Udenfriend's system).

**Method:**

An RP-HPLC-UV-Vis method was developed to separate salicylic acid and isomeric dihydroxybenzoic acids formed in the Udenfriend's system. Confirmation of structures of the oxidized products of salicylic acid, 2,3-dihydroxybenzoic acid and 2,5-dihydroxybenzoic acid was performed by HPLC-ESI-MS/MS method.

**Results:**

The HPLC-UV-Vis method was evaluated for a number of validation characteristics (selectivity, repeatability and intermediate precision, LOD, LOQ and calibration range). It was found that oxidation of salicylic acid resulted in the formation of 2,3- and 2,5-dihydroxybenzoic acids. Furthermore, the hydroxylated metabolites can be further metabolized under the Udenfriend’s conditions.

**Conclusion:**

The results give evidence for possible involvement of the oxidized metabolites of salicylic acid in the development of biological action of salicylates at the site of inflammation, where high hydroxyl radical level can be detected.

## 1 INTRODUCTION

There is an increasing number of lines of epidemiological evidence that reactive oxygen (ROS) and nitrogen (RNS) species are implicated in the pathogenesis of several chronic diseases, such as cancer, cardiovascular and neurodegenerative diseases [1-3]. These reactive species can react with sensitive endogenous and exogenous molecules to form characteristic products. During inflammation, for example, stimulated polymorphonuclear leukocytes (PMN) and macrophages produce large amounts of superoxide ions and hydrogen peroxide [4]. The toxic effects of these oxygen species increase when trace of iron is present, since iron can catalyze formation of hydroxyl radicals [5]. Therefore, importance of non-enzymatic oxidation can be particularly significant under inflammatory conditions. 

A large number of drug molecules (*e.g*. non-steroidal anti-inflammatory agents) containing aromatic rings are administered in the above disorders in which involvement of oxidative damage has been suggested [1-3]. Anti-inflammatory drugs have been reported to scavenge hydroxyl radicals generated in solution at almost diffusion-controlled rates (rate constants about 10^10^ M−^1^ s−^1^) [6]. Based on the latter reactivity, aromatic hydroxylation can be used for measuring *in vitro* hydroxyl radical production [7].

It has been reported that acetylsalicylic acid is rapidly hydrolyzed to salicylate *in vivo* [8]. Formation of 2,3- and 2,5-dihydroxybenzoic acids from salicylic acid has been demonstrated in patients suffering from diseases with increased free radical production and treated by Aspirin [9, 10]. This transformation can also be observed under *in vitro* conditions, so salicylate is a useful tool for measuring *in vitro* hydroxyl radical production and for investigating/modeling non-enzyme-catalyzed hydroxylation of other aromatic compounds [9]. Identification and quantitation of the hydroxylated derivatives have been accomplished by GC [4, 11, 12] and RP-HPLC methods [13-18]. None of the published methods, however, can be applied for separation of the parent salicylic acid and all the isomeric 2,X- dihydroxybenzoic acids in one chromatographic run.

In our present work, Udenfriend’s incubation of salicylic acid (SA) as well as 2,3- and 2,5-dihydroxybenzoic acids (2,3-DHBA and 2,5-DHBA) has been studied by HPLC-UV-Vis method. The developed HPLC method is appropriate to separate and quantitate salicylic acid, all the three isomeric 2,X-dihydroxybenzoic acids as well as 3,4-dihydroxybenzoic acid (3,4-DHBA). Identification of the formed metabolites has been accomplished by reversed phase HPLC-UV-Vis and HPLC-MS methods. Since the halftime of hydroxyl radicals (**∙**OH) is very short, measurement of the secondary products described by the present study may give more complete view related with possible involvement of them in the development of biological action of salicylates.

## 2 MATERIALS AND METHODS

### 2.1 Materials

Salicylic acid (SA), 2,4-dihydroxybenzoic acid (2,4-DHBA) and 3,4-dihydroxybenzoic acid (3,4-DHBA) standards were purchased from Fluka (Budapest, Hungary). 2,3-Dihydroxybenzoic acid (2,3-DHBA) and 2,5-dihydroxybenzoic acid (2,5-DHBA) standards and acetic acid were obtained from Sigma-Aldrich (Budapest, Hungary). LiChrosolv methanol was produced by Merck KGaA (Darmstadt, Germany). Analytical reagent grade diethyl ether and perchloric acid, furthermore puriss grade sodium dihydrogen phosphate (NaH_2_PO_4_, anhydrous) and disodium hydrogen phosphate (Na_2_HPO_4_ x 2 H_2_O) were purchased from Spektrum-3D (Debrecen, Hungary). Analytical reagent grade disodium salt of ethylenediaminetetraacetic acid (Na_2_EDTA), iron(II) ammonium sulphate (Fe(NH_4_)_2_(SO_4_)_2_ x 6 H_2_O) and sodium sulphate (Na_2_SO_4_, anhydrous) were obtained from Reanal (Budapest, Hungary). Analytical reagent grade ascorbic acid was purchased from Molar Chemicals (Budapest, Hungary). Distilled water was purified in the Institute of Pharmaceutical Chemistry, University of Pécs. Mobile phases used for HPLC were degassed in ultrasonic bath (Realsonic cleaner) and filtered through ROBU-GLASS filter before use.

### 2.2 Preparation of Standard Solutions

Stock solutions (5.0 mg/mL) of SA, 2,3-DHBA, 2,4-DHBA, 2,5-DHBA and 3,4-DHBA were prepared in acetonitrile. Working standard solutions were prepared by dilution of the stock solutions with the chromatographic eluent (methanol-acetic acid solution) to give solutions in the concentration range of 2.5-250 μg/mL. Stock solutions as well as working standard solutions were stored at 4 ^o^C. The stock solutions were found to be stable for the 1-month period of the measurements, the working standard solutions were stable only for 7 days. Because of the better stability of the stock solutions acetonitrile was used as solvent. Use of the chromatographic eluent (methanol-acetic acid solution) was necessary during the dilutions, however, because large proportion of acetonitrile in the working standard solutions causes solvent effect. It was found that the acetonitrile-chromatographic eluent volume ratio of the working standard solutions should be at least 1:4.

### 2.3 Chromatographic Systems

#### 2.3.1 HPLC-UV-Vis

The integrated high performance liquid chromatography system (Agilent 1100) was equipped with a quaternary pump, a degasser, an autosampler, an injector with a 100 μL sample loop, a column oven, an ultraviolet-visible and a fluorescent detector. Data were recorded and evaluated using Agilent ChemStation (Rev.B.03.02-SR2) software.

Separation of compounds was performed on a 4 mm × 125 mm, 5 µm particle size, Merck LiChrospher® 60 RP-select B column with guard cartridge (TR-C-160-K1; ABLE Jasco) using isocratic mobile phase consisting of methanol and acetic acid (pH 2.5) (12:88, v/v). The eluent was filtered through glass filter and degassed in ultrasonic bath before use. Chromatography was performed at room temperature. Change in the flow rate was used to shorten the elution time according to the following time schedule (Table **1**).

The peak areas were monitored by a diode-array detector at three wavelengths (235 nm, 247 nm and 258 nm) concerning the absorption maxima of the components to be determined. The injection volume was 25 μL for all sample solutions.

#### 2.3.2 HPLC-ESI-MS

To identify the structure of the formed oxidative metabolites in the Udenfriend’s incubates, a Dionex Ultimate 3000 HPLC system – cross-validated under the same conditions as it is detailed at the Chromatographic system section – connected to a mass spectrometer was used.

Mass spectrometry was performed on a Thermo High Resolution Q Exactive mass spectrometer (Thermo, Bremen, Germany). The Dionex Ultimate 3000 HPLC system was connected to the mass spectrometer with a splitter. Splitting ratio was 1 to 9, where the smaller amount entered the Heated Electrospray Ionization Source II (HESI II) and the larger went to waste. The tray of the autosampler vials was thermostated at 4 °C. The instrument was operated in negative mode. Spray voltage was 3.5 kV, the temperature of the capillary in the HESI was 270 °C. We used nitrogen gas in the source: sheath gas, auxiliary gas, and sweep gas were 40, 8 and 4 arbitrary units respectively. The temperature of the probe heater was set to 30 °C. The RF level of the S-lenses was 70. Data were acquired by alternating scan events: one full scan (m/z 135-175), 3 single ion monitoring (m/z 136.5-138.5, 152.5-154.5, 167.5-171.5), and 3 targeted MS/MS (137.02, 153.02, 169.01 ± 0.95Da). Maximum injection time for full ms scans was set to 200 ms, and for targeted MS/MS 100 ms. The automatic gain control target was set to 3 x 10^6^, and 2 x 10^5^ by MS1 and MS/MS scans respectively.

### 2.4 Method Development

#### 2.4.1 System Suitability

System suitability data were evaluated based on the chromatograms of the solution containing SA, 2,3-DHBA, 2,4-DHBA, 2,5-DHBA and 3,4-DHBA standards (c=5 µg/mL for each component, dissolved in acetonitrile-chromatographic eluent mixture). Evaluation was by relative standard deviation (RSD%) for retention times (t_R_) and peak areas. Results were obtained from 6 parallel injections.

#### 2.4.2 Specificity and selectivity

Specificity tested the ability of the method to differentiate and quantitate the analytes in the presence of endogenous constituents in the sample. The method provides separation and selective determination of SA, 2,3-DHBA, 2,4-DHBA, 2,5-DHBA and 3,4-DHBA in standard solutions. In the course of specificity investigation effect of solvent composition on peak response was also analyzed. It was found that the acetonitrile-chromatographic eluent volume ratio of the working standard solutions should be at least 1:4. If the acetonitrile content of these solutions is higher than recommended solvent effect appears.

#### 2.4.3 Precision

Precision was studied by investigating repeatability and intermediate precision. In order to determine repeatability, solutions containing known amount of salicylate standards were prepared and analyzed. For intra-day data 3 parallel injections of 2 parallel dilutions of 2 independent weightings of solutions containing SA, 2,3-DHBA, 2,4-DHBA, 2,5-DHBA and 3,4-DHBA standards (c=50 µg/mL for each component dissolved in acetonitrile-eluent mixture) were measured.

Intermediate precision was determined by measuring inter-day (by injection of the samples over three consecutive days) data of 3 parallel injections of 3 dilutions (from 2 weightings) of the solution containing SA, 2,3-DHBA, 2,4-DHBA, 2,5-DHBA and 3,4-DHBA (c=50 µg/mL for each component, in acetonitrile-eluent mixture). Evaluation was by relative standard deviation (RSD%).

#### 2.4.4 Linearity

Linearity was studied by preparing standard solutions containing SA, 2,3-DHBA, 2,4-DHBA, 2,5-DHBA and 3,4-DHBA standards at different concentrations from 2.5 to 250 µg/mL in acetonitrile-eluent mixture, plotting a graph of concentration against peak area, and determining the linearity by least-squares regression. Using standard solutions, the method was linear in the range of 2.5-250 µg/mL for all above-mentioned salicylates as well. Data were obtained at 7 levels of concentration (2.5; 5; 10; 25; 50; 100; 250 µg/mL) from 6 parallel injections of 2 independent weightings of the substances.

#### 2.4.5 Limit of Detection (LOD) and Limit of Quantification (LOQ)

The limit of detection (LOD) was determined experimentally, and taken as the concentration producing a detector signal that could be clearly distinguished from the baseline noise (3 times baseline noise). The limit of quantification (LOQ) was taken as the concentration that produced a detector signal ten times greater than the baseline noise [19] by testing salicylate standard solutions.

### 2.5 Udenfriend’s Assay

To a test tube were added 2.0 mL of distilled water, 4.0 mL of 0.1 M phosphate buffer (pH 7.2), 1.0 mL of 10 mM salicylate aqueous solution (SA, 2,3-DHBA, or 2,5-DHBA), 1.0 mL of 10 mM ascorbic acid, 1.0 mL of 2.4 mM Na_2_EDTA and 1.0 mL of 2.0 mM Fe(NH_4_)_2_(SO_4_)_2_ solution, in the order of listing. The mixture was shaken after addition of each component. The mixture was incubated for 30 min or 60 min in a water bath of 37 ^o^C. At the end of the incubation periods the reaction was stopped; 1.0 mL aliquot was taken from the mixture and 1.0 mL of 0.4 M ice-cold perchloric acid was added into it. This acidic solution was cooled down in iced water and kept in it until the extraction started. The samples were extracted two times with 3.0 mL of diethyl ether using separating funnel. The combined ether layers were dried over Na_2_SO_4_ and evaporated under nitrogen. Before HPLC-MS analysis at 247 nm, the dry residue was reconstructed in 100 µL of the mixture of acetonitrile and mobile phase (10:90, v/v).

## 3 RESULTS

### 3.1 Method Development

The method was evaluated in accordance with ICH guidelines [19] for system suitability, specificity and selectivity, precision and linearity.

### 3.2 System Suitability

**Fig. (1):**
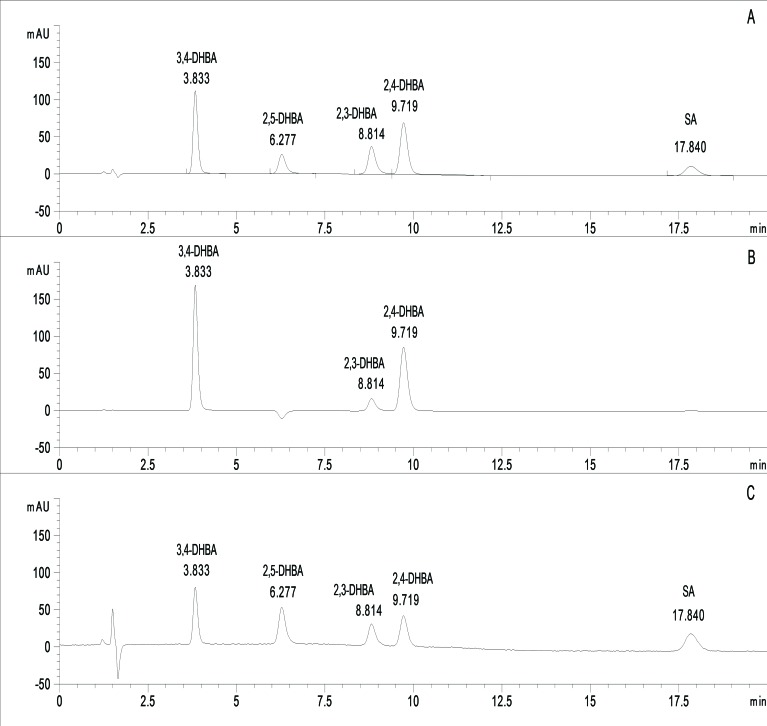
Chromatogram of system suitability solution at different wavelenghts. (A: λ=247 nm, B: λ=258 nm, C: λ=235 nm.).

The peak areas of salicylate standard solutions were monitored by a diode-array detector at three wavelengths (235 nm, 247 nm and 258 nm) concerning the absorption maxima of the components to be determined (Fig. **1**). System suitability data (with the respective RSD% values) are summarized in Table **2**.

### 3.3 Specificity and Selectivity

The method provides separation and selective determination of SA, 2,3-DHBA, 2,4-DHBA, 2,5-DHBA and 3,4-DHBA in standard solutions and in the investigated *in vitro* Udenfriend’s incubations. Results indicated no endogenous peaks at the retention times of the analytes.

In the course of specificity investigation, it was found that the acetonitrile-eluent volume ratio of the standard solutions has to be at least 1:4 to reach the best peak response. Based on this experience reconstruction of the Udenfriend’s extracts was performed by acetonitrile-chromatographic eluent mixture (10:90, v/v).

### 3.4 Precision

Precision was studied by investigating repeatability and intermediate precision. Evaluation was based on relative standard deviation (RSD %) (Table **3**).

### 3.5 Linearity

The calibration plot was linear over the concentration range 2.5-250 µg/mL (n=7). The results are given in Table **4**. The correlation coefficient (r^2^) was >0.999 for each analyte in the investigated concentration range, indicative of good linearity.

### 3.6 Limit of Detection (LOD) and Limit of Quantification (LOQ)

The LOD and LOQ data are given in Table **4**.

### 3.7 Udenfriend’s Assay

**Fig. (2):**
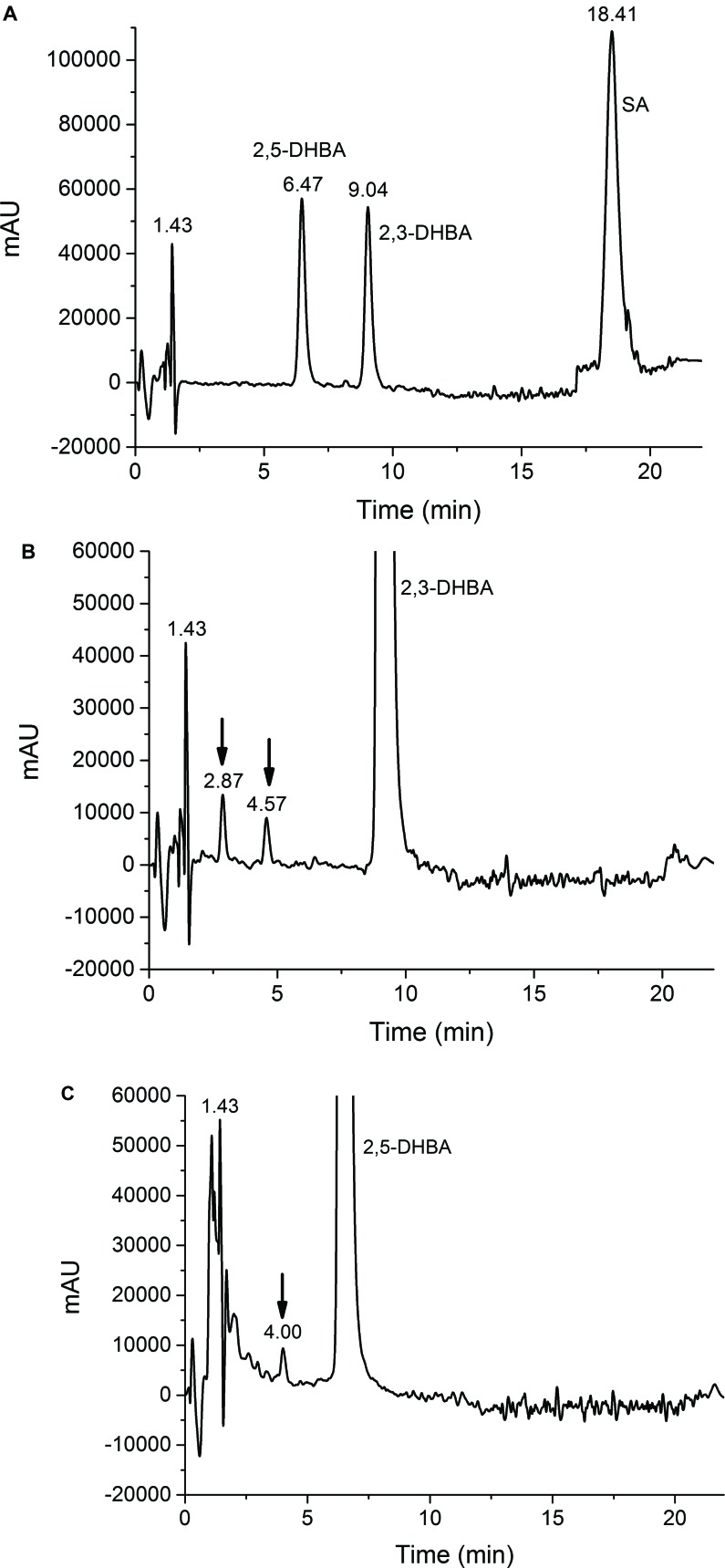
HPLC chromatogram of the Udenfriend’s incubates of salicylic acid (A), 2,3-DHBA (B) and 2,5-DHBA (C). Incubation time was 30 min. Measurements were performed at 247 nm.

**Fig. (3):**
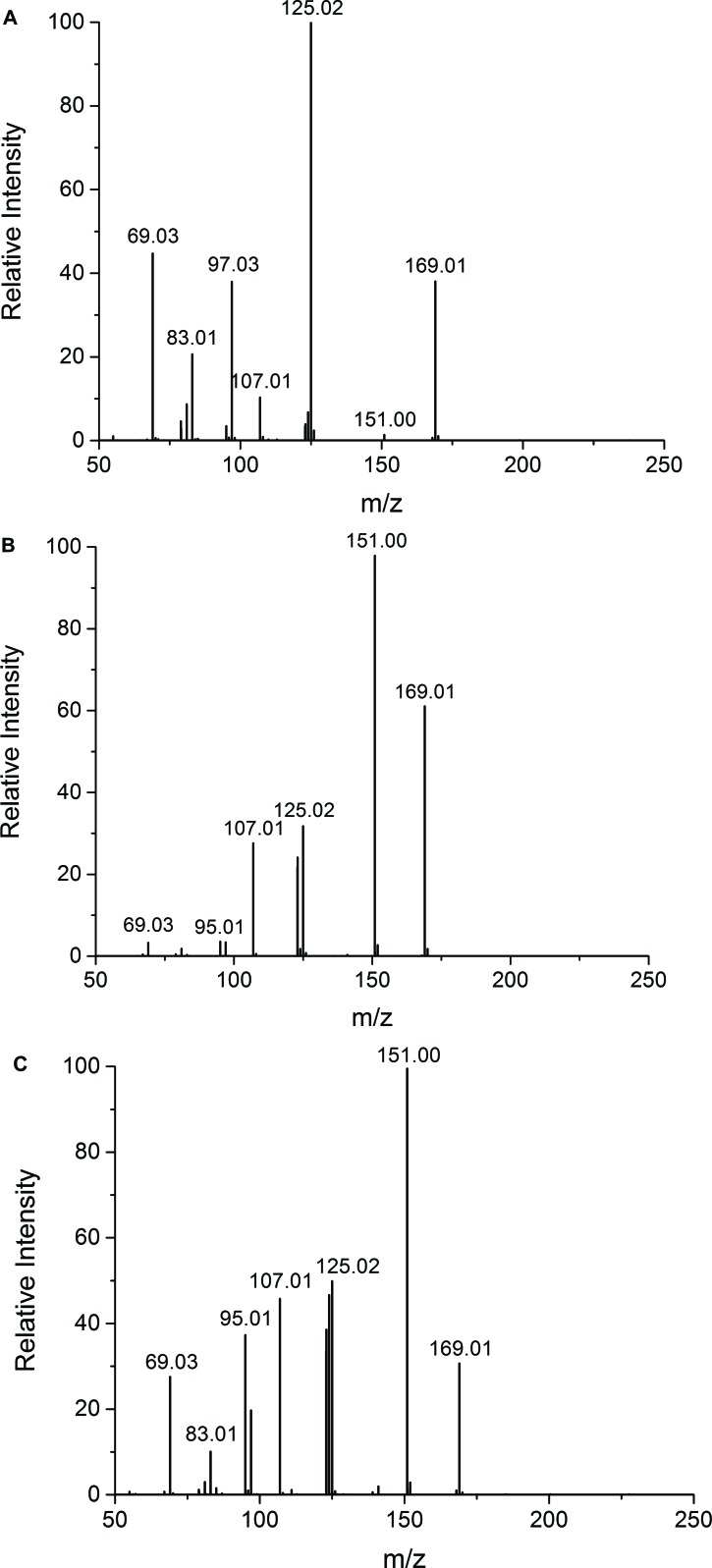
Tandem MS spectra of 2,3-DHBA and 2,5-DHBA. (A): MS/MS spectrum corresponding to the 2.87 min peak of the Udenfriend’s incubate of 2,3-DHBA (MS2 169.01 scan 50-365), (B): MS/MS spectrum corresponding to the 4.57 min peak of the Udenfriend’s incubate of 2,3-DHBA (MS2 169.01 scan 50-365), (C): MS/MS spectrum corresponding to the 4.00 min peak of the Udenfriend’s incubate of 2,5-DHBA (MS2 169.01 scan 50-365).

It was found that the Udenfriend’s incubation of salicylic acid results in formation of 2,3-DHBA and 2,5-DHBA (Fig. **2A**). Furthermore, oxidative metabolites of the same molecular mass could be observed in the Udenfriend’s incubates of 2,3-DHBA and 2,5-DHBA. The structure of the formed metabolites - appearing at 2.87 and 4.57 min, and 4.00 min on the chromatograms of the incubates of 2,3-DHBA and 2,5-DHBA, respectively, (Figs. **2B** and **2C**) - could be identified by ESI-MS/MS as isomeric trihydroxybenzoic acids. The structural similarity of the three metabolites was confirmed by the same molecular mass (169 Da in negative mode) and their very similar mass spectrometric fragmentation patterns showing differences only in the proportion of the fragments (Figs. **3A**-**3C**).

## 4 DISCUSSION

Udenfriend *et al*. described a model system consisting of EDTA, ascorbic acid, molecular oxygen and iron(II) ion in which phenolic compounds are produced as a result of hydroxylation of aromatic rings [20]. This model proved to be a useful method for synthesis of hydroxylated metabolites formed in enzyme-catalyzed oxidation of xenobiotics [21]. Although the mechanism of the reaction is still under debate increasing body of evidence support that the reaction proceeds by a triplet oxygen atom transfer mechanism [22].

In the Udenfriend’s system salicylic acid has been reported to be converted to 2,3- and 2,5-dihydroxybenzoic acids [20, 23]. Catechol was also reported as a product in the *in vivo* salicylate assay [7]. No report has been appeared, however, on the fate of the primary hydroxylated metabolites of salicylic acid under such oxidative conditions. Since oxidative transformation of the dihydroxybenzoic acid metabolites can occur under physiological conditions results of these oxidation reactions are important from both chemical and biological points of view.

Under our modified Udenfriend conditions (pH 7.2; ratio of iron(II)/ Na_2_EDTA = 1.2; ratio of iron(II)/ascorbate = 5) oxidation of salicylic acid resulted in formation of close to equal amount of the expected two (2,3- and 2,5-) dihydroxybenzoic acids. This result calls attention that *in vivo* Udenfriend type non-enzymatic hydroxylation of salicylic acid (*e.g*., involving iron(II), ascorbic acid and citric acid) [24] can modify the generally accepted view that formation of 2,5-DHBA (gentisic acid) is exclusively formed by means of CYP 450-catalyzed oxidation [10].

It was found that the two primary hydroxylated salicylic acid derivatives (2,3- and 2,5-DHBAs) can also react with the Undenfriend reagent. In the extracts of the two incubations three new compounds could be detected that were identified as isomeric trihydroxybenzoic acids by HPLC- ESI-MS/MS. This new finding, draws attention to the possible antioxidant – and prooxidant – effects of the primary DHBA metabolites

## CONCLUSION

A selective RP-HPLC-UV-Vis method was developed to separate salicylic acid and isomeric dihydroxybenzoic acids. The method was used to analyze product composition of oxidation products of SA and 2,3- and 2,5-DHBAs. It was found that the hydroxylated metabolites of salicylic acid (2,5- and 2,3-DHBAs) can be further metabolized under the Udenfriend’s conditions. The results give evidence for possible involvement of the oxidized metabolites in development of biological action of salicylates at the site of inflammation, where high hydroxyl radical level can be detected.

## Figures and Tables

**Table 1:** Time scedule for elution.

**Time (min)**	**Flow rate (mL/min)**
0.00-7.20	1.0
7.20-8.20	1.0-1.5
8.21-11.00	1.5
11.00-12.00	1.5-2.0
12.01-20.00	2.0
20.00-21.00	2.0-1.0

**Table 2:** System suitability data (n=6) of a standard solution containing salicylic acid, 2,3-dihydroxybenzoic acid, 2,4-dihydroxybenzoic acid, 2,5-dihydroxybenzoic acid and 3,4-dihydroxybenzoic acid (5 μg/mL for each component), measured at 247 nm.

**Standards**	**t_R_ (min)**	**RSD% for t_R_**	**Area**	**RSD% for area**	**k**	**Symm**
3,4-DHBA	3.861	2.47	343.53	7.44	1.970	0.80
2,5-DHBA	6.320	2.49	152.32	9.30	3.862	0.77
2,3-DHBA	8.876	0.62	120.57	4.33	5.828	0.75
2,4-DHBA	9.788	1.49	277.28	7.42	6.529	0.83
SA	17.965	1.11	137.28	8.49	12.819	0.83

t_R_: retention time of compound

RSD%: relative standard deviation (%)

k: retention factor

Symm: symmetry factor

DHBA: dihydroxybenzoic acid; SA: salicyclic acid

**Table 3:** System precision data of standard solution (c=50 μg/mL).

**Standards **	**Repeatability**		**Intermediate precision**	
	mean area	RSD%	mean area	RSD%
3,4-DHBA	3199.87	2.638	3297.87	4.921
2,5-DHBA	1438.88	2.196	1476.25	4.176
2,3-DHBA	1431.08	4.782	1468.65	5.322
2,4-DHBA	2664.00	1.289	2696.24	4.448
SA	1194.34	3.095	1242.43	6.277

RSD%: relative standard deviation (%)

DHBA: dihydroxybenzoic acid; SA: salicylic acid

**Table 4:** Data on linearity.

**Standards **	**λ_max_ (nm)**	**Calibration range (μg/mL)**	**Linear equation**	**R^2^**	**LOD (μg/mL)**	**LOQ (μg/mL)**
3,4-DHBA	258	2.5-250	y=63.077x+30.238	0.9999	1.0	2.5
2,5-DHBA	235	2.5-250	y=27.504x+25.623	0.9997	2.0	2.5
2,3-DHBA	247	2.5-250	y=28.555x−41.925	0.9995	2.0	2.5
2,4-DHBA	258	2.5-250	y=55.925x−21.311	0.9998	2.0	2.5
SA	235	2.5-250	y=24.742x−6.336	0.9999	2.0	2.5

R^2^: correlation coefficient

DHBA: dihydroxybenzoic acid; SA: salicylic acid

## References

[1] Halliwell B., Grootveld M. (1987). The measurement of free radical reactions in humans.. FEBS Lett..

[2] Favier A.E., Pierre J-L. (1995). Analysis of Free Radicals in Biological Systems; A. Favier, J. Cadet, B. Kalyanaraman, Marc Fontecave..

[3] Sies H. (1997). Antioxidants in Disease Mechanisms and Therapy.

[4] Richmond R., Halliwell B., Chauhan J., Darbre A. (1981). Superoxide-dependent formation of hydroxyl radicals: Detection of hydroxyl radicals by the hydroxylation of aromatic compounds.. Anal. Biochem..

[5] Winterbourn C.C. (1995). Toxicity of iron and hydrogen peroxide: The Fenton reaction.. Toxicol. Lett..

[6] Hiller K.O., Hodd P.L., Willson R.L. (1983). Antiinflammatory drugs: Protection of a bacterial virus as an *in vitro* biological measure of free radical activity.. Chem. Biol. Interact..

[7] Halliwell B., Kaur H., Ingelman-Sunndberg M. (1991). Hydroxylation of salicylate as an assay for hydroxyl radicals: A cautionary note.. Free Radic. Biol. Med..

[8] Mays D.C., Sharp D.E., Beach C.A., Kershaw R.A., Bianchine J.R., Gerber N. (1984). Improved method for the determination of aspirin and its metabolites in biological fluids by high performance liquid chromatography: Applications to human and animal studies.. J. Chromatogr. A.

[9] Grootveld M., Halliwell B. (1986). Aromatic hydroxylation as a potential measure of hydroxyl-radical formation *in vivo*. Identification of hydroxylated derivatives of salicylate in human body fluids.. Biochem. J..

[10] Ghiselli A., Laurenti O., de Mattia G., Maiani G., Ferro-Luzzi A. (1992). Salicylate hydroxylation as an early marker of *in vivo* oxydative stress in diabetic patients.. Free Radic. Biol. Med..

[11] Luo X., Lehotay D.C. (1997). Determination of hydroxyl radicals using salicylate as a trapping agent by gas chromatography-mass spectrometry.. Clin. Biochem..

[12] Nguyen V., Bonds D.V., Prokai L. (2008). Measurement of hydroxyl-radical formation in the rat striatum by *in vivo* microdialysis and GC-MS.. Chromatographia.

[13] Bakar S.K., Niazi S. (1983). High performance liquid chromatographic determination of aspirin and its metabolites in plasma and urine.. J. Pharm. Sci..

[14] O’Kruk R.J., Adams M.A., Philip R.B. (1984). Rapid and sensitive determination of acetylsalicylic acid and its metabolites using reversed-phase high-performance liquid chromatography.. J. Chromatogr. B.

[15] Grootveld M., Halliwell B. (1988). 2,3-Dihydroxybenzoic acid is a product of human aspirin metabolism.. Biochem. Pharmacol..

[16] Coudray C., Mangournet C., Bouhadjeb S., Faure H., Favier A.J. (1996). The identification of salicylates as normal constituents of serum: a link between diet and health?.. Chrom. Sci..

[17] Yamamoto E., Takakuwa S., Kato T., Asakawa N. (2007). Sensitive determination of aspirin and its metabolites in plasma by LC-UV using on-line solid-phase extraction with methylcellulose-immobilized anion-exchange restricted access media.. J. Chromatogr. B Analyt. Technol. Biomed. Life Sci..

[18] Bektaşoğlu B., Özyürek M., Güçlü K., Apak R. (2008). Hydroxyl radical detection with a salicylate probe using modified CUPRAC spectrophotometry and HPLC.. Talanta.

[19] Bektaşoğlu B., Özyürek M., Güçlü K., Apak R. (2008). Hydroxyl radical detection with a salicylate probe using modified CUPRAC spectrophotometry and HPLC.. Talanta.

[20] (1954). Udenfriend, S.; Clark, C.T.; Axelrod, J.; Brodie, B.B. Ascorbic acid in aromatic hydroxylation. I. A model system for aromatic hydroxylation. J. Biol. Chem., **1954**, 208, 731-740. [20b] Brodie, B.B.; Axelrod, J.; Shore P.A.; Udenfriend, S. Ascorbic acid in aromatic hydroxylation. II. Products formed by reaction of substrates with ascorbic acid, ferrous ion, and oxygen.. J. Biol. Chem..

[21] Slavik R., Peters J-U., Giger R., Bürkler M., Bald E. (2011). Synthesis of potential drug metabolites by a modified Udenfriend reaction.. Tetrahedron Lett..

[22] Hamilton G.A., Hayaishi O. (1974). Molecular Mechanisms of Oxygen Activation..

[23] Coudray C., Talla M., Martin S., Fatome M., Favier A. (1995). high-performance liquid chromatography-electrochemical determination of salicylate hydroxylation products as an *in vivo* marker of oxidative stress.. Anal. Biochem..

[24] Li M., Carlson S., Kinzer J.A., Perpall H.J. (2003). HPLC and LC-MS studies of hydroxylation of phenylalanine as an assay for hydroxyl radicals generated from Udenfriend’s reagent.. Biochem. Biophys. Res. Commun..

